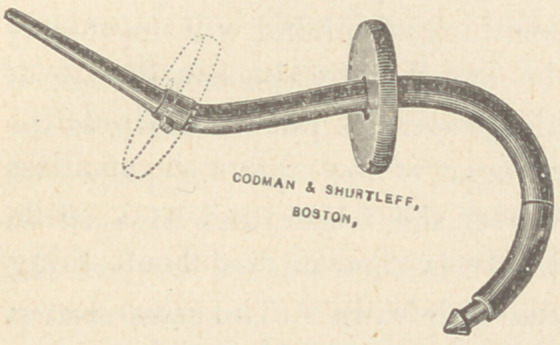# A New Stem Pessary, with Movable Disc

**Published:** 1878-01

**Authors:** E. Cutter

**Affiliations:** Cambridge, Mass.


### A NEW STEM PESSARY WITH MOVABLE DISC.

BY E. CUTTER, M. D., CAMBRIDGE, MASS.

Sometimes it is difficult to introduce a stem pessary into the uterine cavity, because the flexion it is designed to straighten, the narrowness of the os uteri, and the sphincters of the os vaginæ all resist. Sometimes, too, the uterus itself will rise out of reach on the end of the stem, and at times the stem will pocket itself in the uterine wall. It is generally necessary first to reduce the flexion by means of a uterine sound, next to pass the stem through the os by the side of the sound. When the ordinary, or the writer's stem pessary, with fixed disc, is employed in this way, a great obstacle is found in the excessive stretching of the mouth of the womb caused by the triangle made by the sound, the stem and the semi-diameter of the disc. The sound forms one side of the triangle, the stem of the pessary another, and the half disc the base. To properly introduce the stem, the os must be dilated so as to swallow this whole triangle. Sometimes it will do it readily; at others, the point of the stem will not pass beyond the angle of flexion. In this case, if the sound is withdrawn, the stem can be pushed up into place. But if the point of the stem halts before reaching the flexion, and the sound is withdrawn, the stem will rarely be pushed beyond, because the flexion is so readily re-established when the sound is removed.

To obviate the stretching of the os uteri, it would theoretically be the simplest way to get rid of the base and one side of the triangle described above, by passing the stem immediately after the sound is withdrawn. This can rarely be done, as a flexed uterus is very much like a bent steel spring — the flexion will return as soon as the extension is withdrawn. We are left then to pass the stem by the side of the sound, which is buried in the uterine cavity, and which is straightening out the flexion. The base of the triangle described must be reduced to a minimum. This the writer formerly accomplished by slotting the disc down to the axis of the stem. This procedure permits the cylinder of the stem and the sound to lie side by side, and offers the smallest obstacle to introduction. *But* (how often this little word spoils our expectations) it was found practically also a difficult thing to keep the sound in this slot, or prevent the knob (which serves as a mark two and a half inches from the distal end) from becoming engaged and caught in the disc, so that both sound and stem would have to be removed before they could be separated; thus defeating the whole operation.

It was then thought best to have the disc movable — to insert it first of all into the vagina, pass the sound through it, and then pass the stem through that, and so on. It was found, however, that this method would require too large a foramen in the disc, and that the sound would be introduced with greater difficulty than before.

It next occurred to have the disc movable on the cylinder of the writer's stem pessary, towards the proximal end — to pass the denuded stem side by side with the sound — to withdraw the sound and then slip up the disc into its place. This idea, carried out, has been found to be practical. The base of the triangle above referred to, and the necessary stretching out of the os uteri, are reduced to a minimum. The contraction of the vagina and the pressure of the perineum are rendered very much less by the disc being entirely outside the body until the stem is properly introduced, and the whole operation is much simplified. The disc has a central perforation large enough to admit the cylinder of the pessary. On the inside of the circumference of this perforation, an annular channel or square groove is cut. Another groove is cut vertically leading into the annular groove from above. Opposite to this vertical groove, is a small notch in the edge of the disc, which serves to mark its location. A pin is inserted into the base of the stem of the pessary, which fits into the grooves above named. When this pin enters the vertical groove, it passes into that which is annular, and the disc is thus held securely. The chance of its escaping is small when the disc is turned through about half a circle.

The figure represents such a stem pessary. It shows the movable disc in the act of being passed up to the bayonet catch at the base of the stem. The notch is rather too faintly represented in the cut. A small pin inserted in the under surface of the disc answers better than the notch. The dotted lines show the situation of the disc over the pin in its final location. The transverse line between the disc and the proximal end of the pessary, marks the site of a joint, which allows of the end being turned away during the act of defecation, and being held thus supporting the uterus at a time when it is most needed.

These stems must be used with care. It is not enough to send for a stem pessary, and apply it to the first case that comes under observation. A process of preparation should precede its use.

The diagnosis of simple flexion should be assured. There should be no metritis, or perimetritis, nor any complications of diseased conditions.

*The patient should be willing to lie in bed for a week, if necessary.* The general health should be cared for with the proper generous diet and medicine. A compliance with the conditions named above, implies an examination with the uterine sound. If conducted properly, it throws light upon most of the doubtful points. At the same time, the depth of the uterine cavity should be noted, and also the distance from the os uteri to the perineum. A stem pessary should be selected *after due consideration of*

*existing conditions.* Its stem should be one half or one quarter of an inch shorter than the depth of the uterine cavity ; and its cylinder should be half an inch longer than the measurement from the os to the perineum.

When every preliminary is arranged rightly, the patient may recline laterally on the padded top of a dining-table. The left arm should project behind, and the knees be drawn up. The sound may then be introduced, straightening out the flexion, and the patient, resting her right fore arm on her right thigh, should hold it in place ; or an assistant may do the same thing. Unless the fore arm is resting upon something, the hand will insensibly move, and the uterine sound be expelled by the contraction of the uterus. Next the disc of the pessary is pushed down to the proximal end, and the left forefinger of the operator placed on the os, when the stem is passed over the finger, and engaged in the os by the side of the sound. It is then pushed beyond the site of the flexion, and the sound withdrawn. The stem is next pushed into place — a point made known by the position of the pin on the stem-shaft. The disc is then slipped along the cylinder by the left forefinger through the vagina, and its grooves are engaged on the pin. The notch in the disc serves as a guide-point. The facility with which the pin engaged in the grooves has surprised me in every instance of trial. After engagement, the disc is turned about half a circle, and the suspensory cord is held in the hand, while the belt is secured around the waist. If the cord is slack, it should be tightened; if tight, it should be slackened. The hook of the pessary should entirely clear the perineum, and air should circulate between them. If, as sometime happens, sooner or later, the uterus should rise on the pessary, so that it touches the perineum — a longer pessary should be selected. Vaginal examinations should be made from time to time, and if heat, tenderness and throbbing, combined with subjective pain, local, general or sympathetic, are found, *the instrument should be removed.* Indeed, the *patient herself is always instructed to remove the stem if it becomes a source of irritation or pain.*

The instrument is as much under the control of the patient at all times, so far as removal is concerned, as a set of false teeth

When a stem pessary like that described is properly fitted, the flexion of the uterus is reduced, and the organ held in its normal place. The axes of the pessary are those of the normal vagina and uterus. The pessary does not distend the vagina, but merely dilates the canal of the uterus not much beyond the normal measure. From these features the writer has regarded it as theoretically correct. The practical question turns upon its *toleration.*

All cases of flexion will not tolerate a stem; nor will all cases of sickness tolerate medicine. As we do not know beforehand what patients will tolerate medicine, so we do not know what uteri will tolerate stems.

It is not truthful for any one to assert that *no cases whatever* will tolerate a stem pessary. A patient of mine, seen in consultation, lately wore one of my stems for a year with convenience. Others have worn them for months at a time. A stem pessary, introduced just before the menstrual epoch, was worn without trouble. Indeed, the patient said she had not passed so comfortable a period of monthly sickness for several years. There was an especial reason why this procedure was resorted to — it could not be recommended as a general rule.

This is not the occasion for arguing the pros and cons of stem pessaries. It is here simply designed to state that the apparatus here presented has been found to save time, pains and trouble in introduction, and that the device has been worn for long periods of time without trouble. If, after careful attention to the principle which should prevail when fitting body appliances, the device here recommended, should fail, it should be rejected. It is well, however, to remember that the range of its application lies in cases which are not complicated with other diseased conditions, with the exception only of prolapse of the ovaries.

There is another point to be noted with reference to stem pessaries that bear my name. When a patient wears them well, the instrument needs no close attention, because as long as it is worn the flexion is straightened, and the displacement is *cured,* that is *cared* for in the best manner. Displacement rarely occurs. The writer knows of no *fatal* case from its use, and of no very serious trouble from its employment, because of the care always taken to watch the case closely for a few days. If there is increasing trouble indicated by systemic and local signs, the patient is directed to remove the instrument at any time. Those persons who introduce various kinds of stem-pessaries at their offices, and then send their patients home, must expect trouble, for this is tampering with danger. Indeed, the passage of the uterus must, under similar circumstances, have proved a serious matter. But if done where the patient can keep quiet, no harm results.

Singular as it seems, I have a patient who has worn a stem pessary for over a year who could not wear an extra-uterine pessary on account of hyperæsthesia.

A patient suffering with a prolapsed ovary has worn one of my stems for thirty-two months with relief. The idea was to elevate the uterus with this instrument and lift the fallen ovary up into its place. This patient resides in Maine. She went home from Boston wearing the stem, and seventeen months after its introduction the writer examined the uterus with a speculum. There was no infiltration, obstruction or abnormal redness of the cervix. The os contracted down to its usual size. No evidences were presented of the womb having been so long supported on the disc as this is the "pou sto."

Another remarkable thing—this patient occasonally removes the instrument and replaces it. It is not often that it is necessary to remove. She now is wearing a four-inch stem, the longest ever used in my practice. Some years ago, a woman in Maine was bedridden with a uterine flexion. Her physician, at my suggestion, introduced one of my stems, following the directions here indicated. She wore it two years and was entirely cured.

Another woman, similarly situated, a patient of the same physician, also wore one of my stem pessaries for eighteen months, and was entirely relieved.

Four women are now wearing my stems with relief when other means have failed. All these cases were carefully watched to ascertain the degree of toleration. In the light of this experience of several years, I feel justified in resorting to this means of reducing flexions and elevating prolapsed ovaries. Should any one be disposed to pursue the plan, his attention is respectfully invited to the fuller exposition of the writer's opinions and directions contained in a little work recently published by J. Campbell & Son, Boston, entitled, "Uterine Versions and Flexions."

CAMBRIDGE, November, 1877.

## Figures and Tables

**Figure f1:**